# Multi-year patterns in testosterone, cortisol and corticosterone in baleen from adult males of three whale species

**DOI:** 10.1093/conphys/coy049

**Published:** 2018-09-21

**Authors:** Kathleen E Hunt, Nadine S J Lysiak, Cory J D Matthews, Carley Lowe, Alejandro Fernández Ajó, Danielle Dillon, Cornelia Willing, Mads Peter Heide-Jørgensen, Steven H Ferguson, Michael J Moore, C Loren Buck

**Affiliations:** 1 Department of Biological Sciences, Center for Bioengineering Innovation, Northern Arizona University, Flagstaff, AZ, USA; 2 Biology Department, University of Massachusetts Boston, Boston, MA, USA; 3 Arctic Aquatic Research Division, Fisheries and Oceans Canada, Winnipeg, MB, Canada; 4 Department of Biological Sciences, University of Manitoba, Winnipeg, MB, Canada; 5 Department of Birds and Mammals, Greenland Institute of Natural Resources, Nuuk, Greenland; 6 Marine Mammal Center, Woods Hole Oceanographic Institution, Woods Hole, MA, USA

**Keywords:** baleen, glucocorticoids, marine mammals, reproduction, stress, testosterone

## Abstract

Male baleen whales have long been suspected to have annual cycles in testosterone, but due to difficulty in collecting endocrine samples, little direct evidence exists to confirm this hypothesis. Potential influences of stress or adrenal stress hormones (cortisol, corticosterone) on male reproduction have also been difficult to study. Baleen has recently been shown to accumulate steroid hormones during growth, such that a single baleen plate contains a continuous, multi-year retrospective record of the whale’s endocrine history. As a preliminary investigation into potential testosterone cyclicity in male whales and influences of stress, we determined patterns in immunoreactive testosterone, two glucocorticoids (cortisol and corticosterone), and stable-isotope (SI) ratios, across the full length of baleen plates from a bowhead whale (*Balaena mysticetus*), a North Atlantic right whale (*Eubalaena glacialis*) and a blue whale (*Balaenoptera musculus*), all adult males. Baleen was subsampled at 2 cm (bowhead, right) or 1 cm (blue) intervals and hormones were extracted from baleen powder with methanol, followed by quantification of all three hormones using enzyme immunoassays validated for baleen extract of these species. Baleen of all three males contained regularly spaced peaks in testosterone content, with number and spacing of testosterone peaks corresponding well to SI data and to species-specific estimates of annual baleen growth rate. Cortisol and corticosterone exhibited some peaks that co-occurred with testosterone peaks, while other glucocorticoid peaks occurred independent of testosterone peaks. The right whale had unusually high glucocorticoids during a period with a known entanglement in fishing gear and a possible disease episode; in the subsequent year, testosterone was unusually low. Further study of baleen testosterone patterns in male whales could help clarify conservation- and management-related questions such as age of sexual maturity, location and season of breeding, and the potential effect of anthropogenic and natural stressors on male testosterone cycles.

## Teaser

Baleen from three adult male whales (North Atlantic right, bowhead and blue) contains regularly spaced areas of high testosterone content suggestive of annual testosterone cycles. Patterns in glucocorticoids (cortisol and corticosterone) show potential relationships with breeding-related stress as well as with entanglement in fishing gear and possible disease episodes.

## Introduction

Most mysticetes (baleen whales, *Mysticeti*) reproduce seasonally, yet it is unclear whether they also show seasonal patterns in reproductive hormones or in the adrenal glucocorticoids. The annual cycle of a mysticete whale typically involves a migration between polar or subpolar ‘feeding grounds’ in summer months and subtropical ‘breeding grounds’ in winter months, with females typically producing calves only during a calving season in a portion of the winter months (Lockyer and Brown, 1981). These life history features imply seasonal reproduction, i.e. regular alternation between a reproductively active and a reproductively inactive (non-breeding) state (Bronson, 1991).

Seasonal reproduction in other mammals almost universally involves pronounced annual cycles in the reproductive hormones. Typically, changes in photoperiod or other environmental cues signal the approach of the breeding season, and these cues (along with, in some mammals, endogenously generated circannual cycles) stimulate the hypothalamus to release gonadotropin releasing hormone (GnRH), with resultant increases in pituitary hormones (luteinizing hormone, LH, and follicle-stimulating hormone, FSH), which then stimulate seasonal gametogenesis and seasonal secretion of gonadal hormones in both sexes (e.g. testosterone in males, 17β-estradiol in females; Norris and Lopez, 2011). In males, seasonal testosterone patterns have been well-documented in many terrestrial mammals as well as pinnipeds and even several odontocete (toothed) whales (Wells, 1984; Kellar *et al.*, 2009; Funasaka *et al.*, 2011, 2018; O’Brien *et al.*, 2016; Richard *et al.*, 2017). However, cetaceans are cryptic species that are difficult to observe via traditional techniques, and seasonal patterns in many large whales are still incompletely understood, particularly the mysticete whales. A better understanding of cyclic changes in reproductive hormones in these species could improve population management and conservation. For example, a clear understanding of male testosterone cycling in a given species can potentially improve accuracy of sex identification of unknown individuals via hormone ratios, more accurate discrimination of sexually active adults from juveniles, better understanding of age of sexual maturity (often difficult to determine in males), potential occurrence of reproductive senescence in older males, and could help determine month and location of the conceptive season—which in turn may also inform estimates of gestation length of females.

In the mysticete whales, however, it has been difficult to obtain endocrine samples to ascertain whether males do have annual cycles of testosterone. Historic anatomical studies from the era of commercial whaling suggest an annual cycle in testis development, i.e. regular seasonal patterns of testis regression and recrudescence occur in several species of mysticetes (reviewed in Chittleborough, 1955; see also Best, 1984; Mogoe *et al.*, 2000). More recently, some plasma hormone data from lethal sampling of mysticetes have been published, but these data are limited to only certain months (the ‘hunting season,’ when whales are on high-latitude summer feeding grounds) and thus usually exclude the winter breeding season. Some of these latter studies have documented what appears to be the beginning of a rise in testosterone near the end of the summer hunting season (fin, sei and common minke whale [*Balaenoptera (B.) physalus, B. borealis*, *B. acutorostrata*, respectively]; Kjeld, 2001; 2003; Kjeld *et al.*, 2004) while other researchers have reported no difference in testosterone with month (common minke, Bryde’s whale [*B. brydei*]; Watanabe *et al.*, 2004). Since whales were only sampled across a portion of the year in these plasma-based studies, few conclusions can be drawn about the overall annual cycle, a regrettable lack of information given the paucity of data on reproductive cycles in most of these species.

Recently, endocrine analyses of alternative sample types have reinvigorated interest in determining the nature of reproductive cycles in both male and female mysticetes. Fecal hormone analysis has proven informative for studies of pregnancy, lactation and the effects of various anthropogenic stressors (Rolland *et al.*, 2005, 2012, 2017; Hunt *et al.*, 2006; Burgess *et al.*, 2018), but the question of potential annual cycles has remained elusive since fecal samples are often not obtainable on the breeding grounds due to seasonal fasting, and, in some species, the location of the breeding grounds is still unclear (e.g. North Atlantic right whale, *Eubalaena glacialis* [‘NARW’]). Similarly, quantification of hormones in respiratory vapor shows promise for repeated sampling of individual whales over time, but so far the only such data from mysticetes are also limited to spring and summer feeding grounds when mysticetes tend to be more easily approached (Hogg *et al.*, 2009; Burgess *et al.*, 2018). Mysticete ear plugs, obtained postmortem from stranded carcasses, have the advantage of containing lifetime records of steroid hormones that are deposited in consecutive layers of cerumen (earwax), but temporal resolution has proven to be relatively coarse; this tissue type can identify age of sexual maturity but cannot readily discriminate seasonal changes within a year (Trumble *et al.*, 2013). A recent study on humpback whales (*Megaptera novaeangliae*) using blubber samples from biopsy darts did document a significant seasonal change in blubber testosterone, but most animals could be sampled only once and the study covered only one year (Vu *et al.*, 2015); no other male mysticetes have yet been studied with this method. In short, though many of the above techniques have proven effective for identifying reproductive state of females (e.g. Rolland *et al.*, 2005; Kellar *et al.*, 2013; Burgess *et al.*, 2018), endocrine patterns of male mysticetes still remain almost entirely obscure (Pomeroy, 2011).

A related question of interest is the potential effect of natural and anthropogenic stressors on male reproduction. In vertebrates, circulating concentrations of the adrenal glucocorticoids (GCs, aka ‘stress hormones’—cortisol and corticosterone) increase during most forms of stress (Romero and Wingfield, 2015). In seasonally breeding male mammals, baseline GCs often show a predictable increase that directly parallels the seasonal increase in testosterone, presumably in response to breeding-related stress (e.g. energetically demanding courtship for females, aggressive interactions with other males; Romero, 2002; Romero and Wingfield, 2015). Such predictable increases in glucocorticoids during the breeding season do not seem to inhibit reproduction (though there can be individual exceptions, e.g. younger or subordinate males; Frasier *et al.*, 2007; see Romero, 2002; Romero and Wingfield, 2015 for reviews). However, additional unpredictable stressors (e.g. injury, illness, severe weather) can result in higher GC concentrations that trigger what has been termed an ‘emergency life history state,’ during which high GCs directly inhibit gonadal activity (Romero and Wingfield, 2015). For example, an injured, ill, or socially-stressed male mammal may have unusually low testosterone, presumably an adaptive response that represents tradeoffs among current survival and health, current reproduction, and potential future reproductive effort (Wingfield and Sapolsky, 2003; Romero and Wingfield, 2015). Thus, relationships of GCs to testosterone can change from positive to negative correlations with increasing occurrence of unpredictable, non-breeding-related stressors, or with prolonged chronic stress (Romero and Wingfield, 2015). A method to track GCs and testosterone in the same individuals over time could therefore prove invaluable for elucidating long-term patterns in reproduction and stress over years. The only relevant data from male mysticetes is one study reporting a positive correlation of fecal GCs with fecal androgens in male NARW sampled in August and September (Hunt *et al.*, 2006), but the rest of the annual cycle remains unstudied.

Baleen may prove ideal for such questions of long-term patterns in the reproductive and adrenal hormones (Hunt *et al.*, 2014b, 2016). In mysticete whales, keratinous baleen plates are suspended from the upper jaw; these plates grow continuously at a species- and age-specific baleen growth rate (BGR), simultaneously wearing away steadily at the distal (lower) tip (St. Aubin *et al.*, 1984; Mitani *et al.*, 2006; Lubetkin *et al.*, 2008, 2012; Lysiak *et al.*, 2018; Ryan *et al.*, 2013). Our recent studies indicate that all classes of steroid and thyroid hormones are detectable in baleen from at least eight species of mysticete whales (Hunt *et al.*, 2017b), suggesting that hormones are routinely deposited in growing baleen much as they are in growing mammalian hair (Meyer and Novak, 2012; Cattet *et al.*, 2017; Mesarcova *et al.*, 2017; Yamanashi, 2018). Further, the hormones appear to be deposited in linear fashion as the baleen grows, such that a complete baleen plate contains a continuous retrospective record of the whale’s endocrine history spanning the time period of baleen growth (a decade or more in right and bowhead whales, ~3–5 years in most other large mysticetes; St. Aubin *et al.*, 1984; Mitani *et al.*, 2006; Lubetkin *et al.*, 2008, 2012; Lysiak *et al.*, 2018; Bentaleb *et al.*, 2011; Ryan *et al.*, 2013; Eisenmann *et al.*, 2016; Busquets-Vass *et al.*, 2017). For example, in female bowheads (*Balaena mysticetus*) and NARW, baleen progesterone content is dramatically elevated in regions of baleen that were grown during documented pregnancies (Hunt *et al.*, 2014, 2016; Lysiak *et al.*, 2018). Similarly, baleen cortisol and corticosterone are elevated in baleen grown during late pregnancy, and are also strongly elevated in baleen grown during known entanglements in fishing gear and during chronic illness (Hunt *et al.*, 2017a; Lysiak *et al.*, 2018).

In this first study of baleen hormones in male whales, we studied patterns in baleen testosterone and glucocorticoids along the full length of a single adult plate from each of three whale species: bowhead whales, which have longest baleen; NARW, with next-longest baleen; and blue whale (*B. musculus*, ‘blue’), selected as a representative of the rorquals (*Balaenopteridae*) which all have relatively short baleen. Ratios of stable isotopes (SI) of carbon and nitrogen were also analysed as independent confirmation of annual cyclicity. In bowhead and NARW, these SI ratios typically demonstrate regular annual cycles due to seasonal prey-switching between food sources in isotopically distinct summer and winter feeding grounds (Best and Schell, 1996; Lee *et al.*, 2005; Lysiak, 2008; Matthews and Ferguson, 2015; Lysiak *et al.*, 2018). SI ratios were also analysed for the blue whale, though in North Pacific blue whales these SI cycles may be weak or absent due to isotopically similar prey across the year (Busquets-Vass *et al.*, 2017). One of the three individuals, the NARW, was known to have experienced an entanglement and a likely disease episode during the time period of baleen growth (see Methods). Our goals were to determine (1) whether the baleen has regularly spaced testosterone peaks along its length; (2) if so, whether the spacing of these peaks corresponds with estimates of annual BGR and/or with annual cycles in SI ratios (Lubetkin *et al.*, 2008; Lysiak, 2008), i.e. could the patterns represent annual testosterone cycles; (3) whether there is any relationship of baleen glucocorticoids to baleen testosterone, particularly regarding potential impacts of annually predictable breeding-related stressors as opposed to unpredictable stressors such as entanglement and disease.

## Methods

### Baleen samples

Details of the three individual whales in this study are presented in Table 1. The NARW was a known individual (Eg 1238) and was classed as adult based on size at first sighting in 1982; this whale died 19 years later in 2001. Age of sexual maturity in male NARW is thought to be ~9 years (though confirmed paternities do not typically occur until ~15 years; Frasier *et al.*, 2007; Kraus *et al.*, 2007), so this whale was tentatively assigned a minimum age of 28 years. The other two whales were classed as adult based on snout-fluke body length (Table 1), as compared to the following estimates of length at sexual maturity for males: bowhead, 12–13 m (Nerini *et al.*, 1984); blue, 20–21 m for Northern Hemisphere individuals (Sears and Perrin, 2009). Additionally, the bowhead’s age was previously estimated via analysis of aspartic acid racemization of the eye lens, yielding an estimated age of 44 years (bowhead ‘322’ in Table 1 of Heide-Jørgensen *et al.*, 2012), well past the estimated age at sexual maturity of 25 years in bowheads (George *et al.*, 1999). Cause of death was acute for the bowhead and NARW; the bowhead was a presumed-healthy individual harvested during a legal Inuit subsistence hunt in Disko Bay, Greenland, while the NARW died acutely in fishing gear (Moore *et al.*, 2004). Cause of death is not known for the blue whale, which was found dead on the coast of California. All three plates were collected and cleaned of soft tissue at the time of necropsy (Table 1), and were stored at room temperature between the time of collection and hormone assays in 2016–17. Prior studies indicate that steroid hormones are likely stable for at least three decades in baleen stored at room temperature (Hunt *et al.*, 2016, 2017b). Note that initial processing and SI analyses occurred prior to this study in three different laboratories, i.e. the three locations that initially processed the specimens, with subsamples later assayed for hormones in a single laboratory. This resulted in minor methodological differences in sample processing, as detailed below. Data were analysed within-subject only; that is, data were not compared across laboratories.

**Table 1:** Information known for the three adult male whales in this study. NARW = North Atlantic right whale

Species	Individual identity	Curating institution^a^	Collection date	Body length (m)	Baleen length (m)	Cause of death	Other pertinent information
Bowhead	BH3	DFO	30 April 2009	14.1	2.04	Subsistence harvest	n/a
NARW	Eg 1238	WHOI	01 November 2001	14.6	1.24	Acute death in fishing gear	Scarring indicates entanglement in 1998 and possible disease in 1997–98
Blue	CAS MAM 23130	CAS	04 September 1989	23.0	0.65	Unknown	n/a

^a^CAS, California Academy of Sciences; DFO, Fisheries & Oceans Canada; WHOI, Woods Hole Oceanographic Institution.

The NARW, Eg 1238, had been photographed repeatedly in the years prior to death. Sighting records from the North Atlantic Right Whale Sightings and Identification Databases (www.rwcatalog.neaq.org) indicate appearance of at least two types of scars likely indicative of prior stress events. At some point during 1997–98, this whale acquired white circular lesion-like scars on its head. A large proportion of the NARW population developed similar lesions during the same years, during which calving rate also declined sharply and the population exhibited a ‘steep deterioration’ in visible indicators of health (Rolland *et al.*, 2016). These patterns have been interpreted as evidence of a disease epidemic (Hamilton and Marx, 2005). Separately, at some point in 1998, Eg 1238 acquired linear white scars indicative of a ‘minor entanglement’ in fishing gear (P. Hamilton, pers. comm.), with ‘minor’ defined as injuries that were less than 2 cm in width and did not appear to penetrate into blubber (following Knowlton *et al.*, 2012, 2015). Both of these events occurred during the time-period of growth of Eg 1238’s baleen plate.

### Baleen subsampling

Baleen plates were processed as in Hunt *et al.* (2016, 2017a,b) with minor inter-laboratory differences in protocol as follows. The bowhead plate was first cleaned of algae and other adhering material with water and scrubbing pads, followed later by scraping with scalpel blades to remove any additional adhering surface material. This plate was cut at the gumline, and thus did not include the very most recently grown baleen in the root of the plate (~20–26 cm, or ~1.5 years, of growth; Hunt, unpublished data). The NARW and blue whale plates, which did include the root of the plate, were cleaned at the time of necropsy, and were wiped again with 70% isopropyl alcohol prior to sampling. After air-drying, measuring tape was affixed to the labial edge of the concave face of each plate, with the tape following the natural curve of the plate. Sampling intervals (2 cm for bowhead and NARW, 1 cm for blue) were marked along this tape down the entire length of the plate, which remained affixed to the plate for the duration of sampling. The ‘zero’ point (aka ‘base,’ i.e. most recently grown baleen) was defined as the proximal-most (i.e. dorsal-most) point of the baleen (gumline for bowhead, root of the plate for NARW and blue), with all subsequent points measured from the zero point.

Following our previously published protocols (Matthews and Ferguson, 2015; Hunt *et al.*, 2016, 2017a, b), subsamples of baleen powder were collected using a hand-held rotary tool fitted with either a 1/16 inch drill bit (bowhead) or a tungsten-carbide ball tip (NARW, blue) to drill a series of small transverse grooves at the prescribed sampling intervals. In bowhead and NARW, 2 cm intervals are commonly used in SI studies and produce a temporal resolution of an estimated ~30 d per sampling interval in adult NARW baleen and ~40 d per sampling interval in adult bowhead (Lubetkin *et al.*, 2008; Lysiak, 2008). The blue whale plate was sampled at more frequent intervals (1-cm spacing) because no estimates of BGR were yet available for this species; new research indicates an average BGR of ~24 days per 1 cm for adults of this species (Busquets-Vass *et al.*, 2017). Plates were drilled individually, with considerable care taken to avoid potential cross-contamination between subsampling locations on the same plate. Baleen powder from each sampling location was collected on a weigh paper below the plate, mixed well with a metal spatula, and stored in individual vials or bags until further processing.

### Hormone extraction

Hormones were extracted from powdered baleen samples with modifications of Hunt *et al.* (2014, 2016, 2017a, b) as follows: 100% HPLC-grade methanol was added to weighed-out aliquots of baleen powder in 16 × 100 mm borosilicate glass tubes, initially with a ratio of 100 mg powder: 6.00 mL methanol (bowhead) and later in a ratio of 75 mg: 4.00 mL methanol (NARW, blue), after testing confirmed that this change caused no significant differences in hormone concentrations (data not shown). Tubes were capped and vortexed 2 h (Glas-Col Large Capacity Mixer, speed set on 40; Glas-Col, Terre Haute, IN) and centrifuged for 15 min at 3000 g (bowhead), or 5 min at 4025 g (NARW, blue). After centrifugation, 4.00 mL (bowhead) or 3.00 mL (NARW, blue) of methanol supernatant, containing hormones, was transferred to a borosilicate tube for dry-down. Not all supernatant could be recovered because some methanol soaks into the baleen powder; assay results were later corrected for percentage of supernatant recovered. Supernatants were dried either in a warm water bath under N_2_ for ~6 h (bowhead), or overnight in a rotary evaporator under vacuum (NARW, blue). Dried bowhead hormone extracts were then shipped to Northern Arizona University (Flagstaff, AZ, USA) to join the NARW and blue samples; all subsequent laboratory work (i.e. reconstitution and assay) for all three species occurred at the same laboratory. Dried samples were reconstituted in 500 uL of EIA assay buffer (buffer ‘X065,’ Arbor Assays, Ann Arbor, MI, USA), sonicated 5 min, vortexed 5 min, pipetted to a cryovial and left to sit at 4°C for 30 min (allowing remaining particulates to settle out), and finally decanted to an externally threaded, vaporproof, *o*-ring-capped cryovial for long-term storage at −80°C. This is termed the ‘1:1’ (full-strength, neat) extract. All extracts were assayed within four months of extraction.

### Hormone assays

Samples were assayed with testosterone, cortisol and corticosterone enzyme immunoassays that have previously passed parallelism and accuracy validations for all three species (testosterone kit #K032, cortisol kit #K003, and corticosterone kit #K014, Arbor Assays, Ann Arbor, MI, USA; Hunt *et al.*, 2017b), with the exception of corticosterone accuracy validations for bowhead and blue whale, which were performed here. Corticosterone accuracy validations involved assay of a set of standards spiked with pooled 1:1 baleen extract from the bowhead and blue whale, run alongside a set of standards spiked only with buffer. A graph of observed vs. expected dose should produce a straight line with a slope close to 1.0, indicating accurate discrimination of low from high doses at the given sample dilution, even in the presence of unusual sample matrix.

Following successful validations, all samples were analysed for immunoreactive testosterone, cortisol and corticosterone. Samples were assayed at 1:1 in the cortisol and corticosterone assays but were diluted to 1:4 for testosterone assay, so as to keep results as near as possible to 50% bound on the standard curve, the area of greatest assay precision. Each whale’s samples were assayed together with the order of samples randomized within and across assays, to minimize any influences of intra- or inter-assay variation on longitudinal data. The manufacturer’s assay protocols were followed with two modifications: (1) the cortisol standards were brought up in the same X065 buffer used for the other two assays (based on technical advice from the manufacturer); (2) all three assays included an additional low-dose standard, produced by mixing equal volumes of the lowest standard with X065 assay buffer. All assays followed standard QA/QC including assay in quadruplicate of all nonspecific binding wells and blanks (zero dose); assay in duplicate of standards and all samples; re-assay of any sample with coefficient of variation (CV) > 10% between wells; and exclusion of any single standard with > 10% CV from the standard curve. See Hunt *et al.* (2017b) for additional assay details including antibody sources, inter- and intra-assay precision, sensitivity and cross-reactivities. All assay results were converted to nanograms of immunoreactive hormone per g of baleen powder.

### Stable-isotope analysis

For all individuals, 1.0 ± 0.2 mg of baleen powder was weighed directly into tin capsules before δ^15^N and δ^13^C analysis. Bowhead SI analysis employed a Vario EL III elemental analyzer (Elementar, Germany) interfaced with a DELTAplus XP isotope ratio mass spectrometer (Thermo-Fisher Scientific, USA) at the G.G. Hatch Stable Isotope Laboratory (isotope.uottawa.ca; University of Ottawa, Ontario, Canada). NARW SI analysis used a PDZ Europa ANCA-GSL elemental analyzer interfaced with a PDZ Europa 20-20 isotope ratio mass spectrometer (Sercon Ltd, Cheshire, UK) at the University of California Stable Isotope Facility (stableisotopefacility.ucdavis.edu; University of California, Davis, CA, USA). Finally, blue whale SI analysis employed a Carlo Erba NC2100 elemental analyzer (MFR) interfaced with a Thermo-Electron Delta V Advantage isotope ratio mass spectrometer configured through a Finnigan CONFLO III at the Colorado Plateau Stable Isotope Laboratory (isotope.nau.edu; Northern Arizona University, Flagstaff, AZ, USA). All three laboratories employ normalization to international reference standards; for additional details for bowhead and NARW analyses, see Matthews and Ferguson (2015) for bowhead, and Lysiak *et al.* (2018) for NARW. For the blue whale, NIST (National Institute of Standards and Technology, USA) standard 1547 (peach leaves) was interspersed throughout the run every ~11 samples to check for intra-run drift, and all data were normalized in reference to the calibrated IAEA (International Atomic Energy Association) reference standards CH6, CH7, N1 and N2, with additional elemental correction standards (i.e. known %C and %N values; acetanilide, BBOT, cyclohexanone, cysteine, methionine, nicotinamide and sulfanilamide) and secondary check (‘unknown’) standards (caffeine, NIST apple leaves, NIST bovine liver, NIST mussel tissue, NIST pine needles, NIST tomato leaves) included in the run in quadruplicate. SI ratios for all whales are reported in delta notation (*δ*, in parts per thousand) as the ratio of unknown sample to an international standard (atmospheric N2 and Vienna Pee-Pee Belemnite limestone for δ^15^N and δ^13^C, respectively). As δ^15^N has been reported to have more pronounced and predictable annual variation in all three species than does δ^13^C, analysis of hormonal periodicity focused on δ^15^N (Best and Schell, 1996; Lee *et al.*, 2005; Lysiak, 2008; Matthews and Ferguson, 2015; Lysiak *et al.*, 2018; Busquets-Vass *et al.*, 2017). However, δ^13^C data are also presented for comparison to previous literature. SI data were also consulted to verify that individual BGR was approximately similar to published species-specific estimates of BGR derived from other specimens, e.g. species-specific averages of ~17–18 cm/year for adult bowhead (Lubetkin *et al.*, 2008; Matthews and Ferguson, 2015), ~24 cm/year for adult NARW (Lysiak, 2008) and ~16 cm/year for adult blue whale (Busquets-Vass *et al.*, 2017).

### Statistical analysis

Accuracy results for the corticosterone assay for bowhead and blue whale were assessed with linear regression of observed dose versus known standard dose, with acceptable accuracy defined as *r*^2^ > 0.95, no deviation from linearity (runs test *P* > 0.05), slope within 0.7–1.3 (ideal slope = 1.0) and *y*-intercept of the regression line within 50% of the pool when assayed alone (Grotjan and Keel, 1996; Ezan and Grassi, 2000). Potential periodicity of testosterone, cortisol, corticosterone, δ^15^N, and δ^13^C were assessed using autocorrelation analysis of each whale’s data separately, with period estimated from spectra of autoregressive models fit to detrended data. For whales in which detrended δ^15^N exhibited significant periodicity within the range of published species-specific BGR (i.e. whale exhibits likely annual SI cycles), detrended hormone data were tested for correlations with detrended δ^15^N, to examine whether hormone periodicity might also represent annual cycles. Detrended δ^15^N and detrended δ^13^C were also tested for correlations with each other, for additional verification that δ^15^N patterns likely represent annual cyclicity. For each whale, the three hormones were also tested for correlations with each other; this analysis employed raw (non-detrended) data, since multi-year trends over time can be biologically meaningful for steroid hormones. Maximum and minimum testosterone concentrations for each successive testosterone period along each baleen plate were then calculated using each individual whale’s period length estimated by autocorrelation analysis, and average peak amplitude was calculated as [average testosterone maximum]/[average testosterone minimum]. As a preliminary investigation into the reported mild slowing of BGR as whales age (Lubetkin *et al.*, 2008), average inter-peak interval was also calculated separately for the older half of the plate and the newer half of the plate, for any whales that had three or more testosterone peaks in each half of the plate (i.e. six or more peaks in total across the full plate). Accuracy analyses, correlations, and maxima/minima calculations were performed with Prism 7.0c for Macintosh; autocorrelations for periodicity analysis were performed with JMP 14. Two-tailed tests were employed for all analyses with a significance threshold of 0.05.

## Results

### Corticosterone assay accuracy

Accuracy was acceptable for the corticosterone assay for both bowhead and blue whale baleen extract, with *r*^*2*^> 0.95 for both species (bowhead, *r*^*2*^ = 0.9985; blue, *r*^*2*^ = 0.9876), no deviation from linearity (bowhead, *P* = 0.2000; blue, *P* = 0.8000), slopes within the desired range of 0.7–1.3 (bowhead, slope = 1.059; blue, slope = 1.279), and y-intercept within 50% of pooled extract when assayed alone (bowhead, y-intercept = 181 pg/mL, pool = 141 pg/mL; blue whale, y-intercept = 339 pg/mL, pool = 210 pg/mL).

### Testosterone

**Figure 1:**
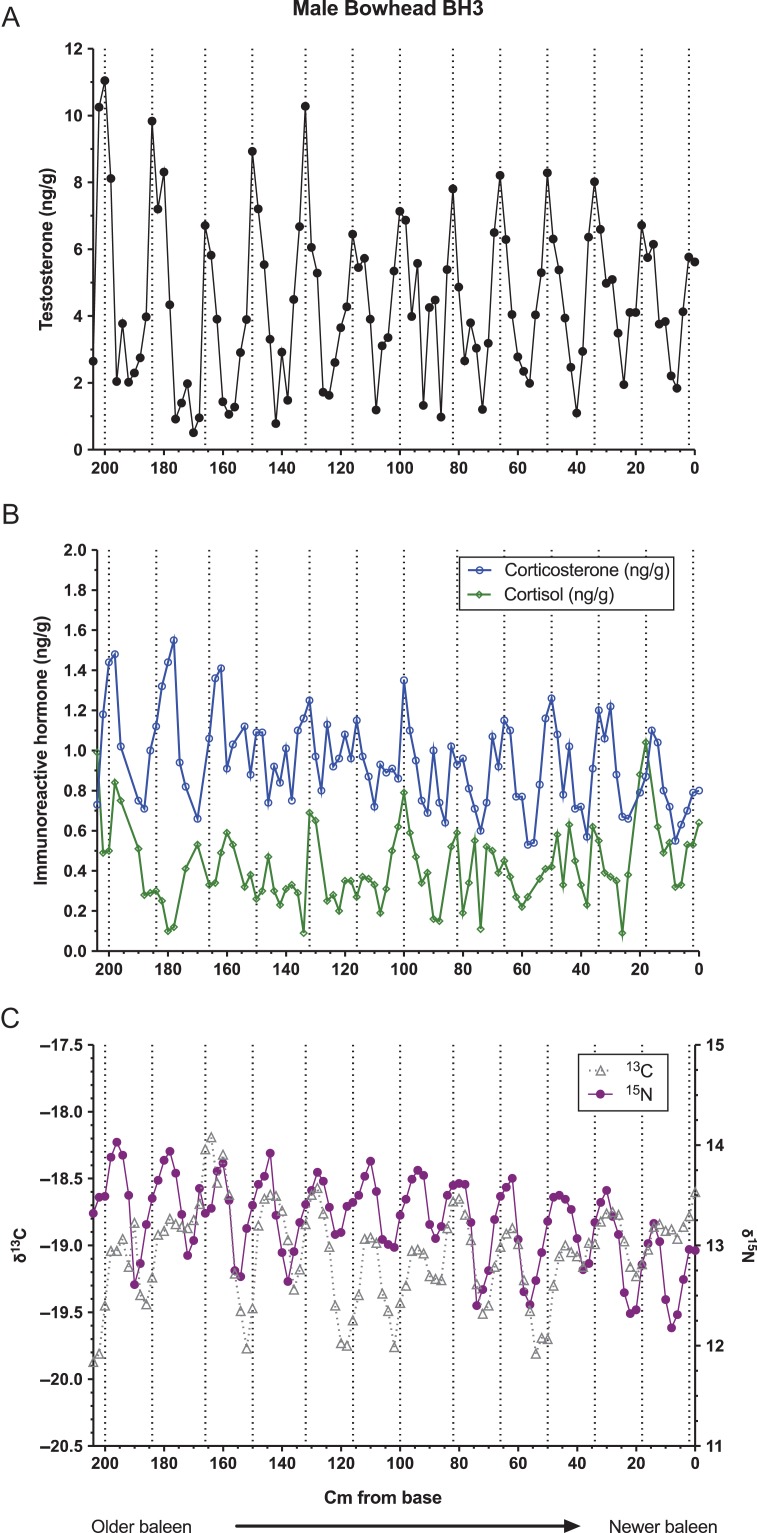
Patterns in concentrations of immunoreactive testosterone (top, A), glucocorticoids (middle, B), and ratios of stable isotopes of nitrogen and carbon (bottom, C) along the full length of a baleen plate from a single adult male bowhead whale, individual BH3. Dotted lines indicate locations of local testosterone maxima and minima.

**Figure 2:**
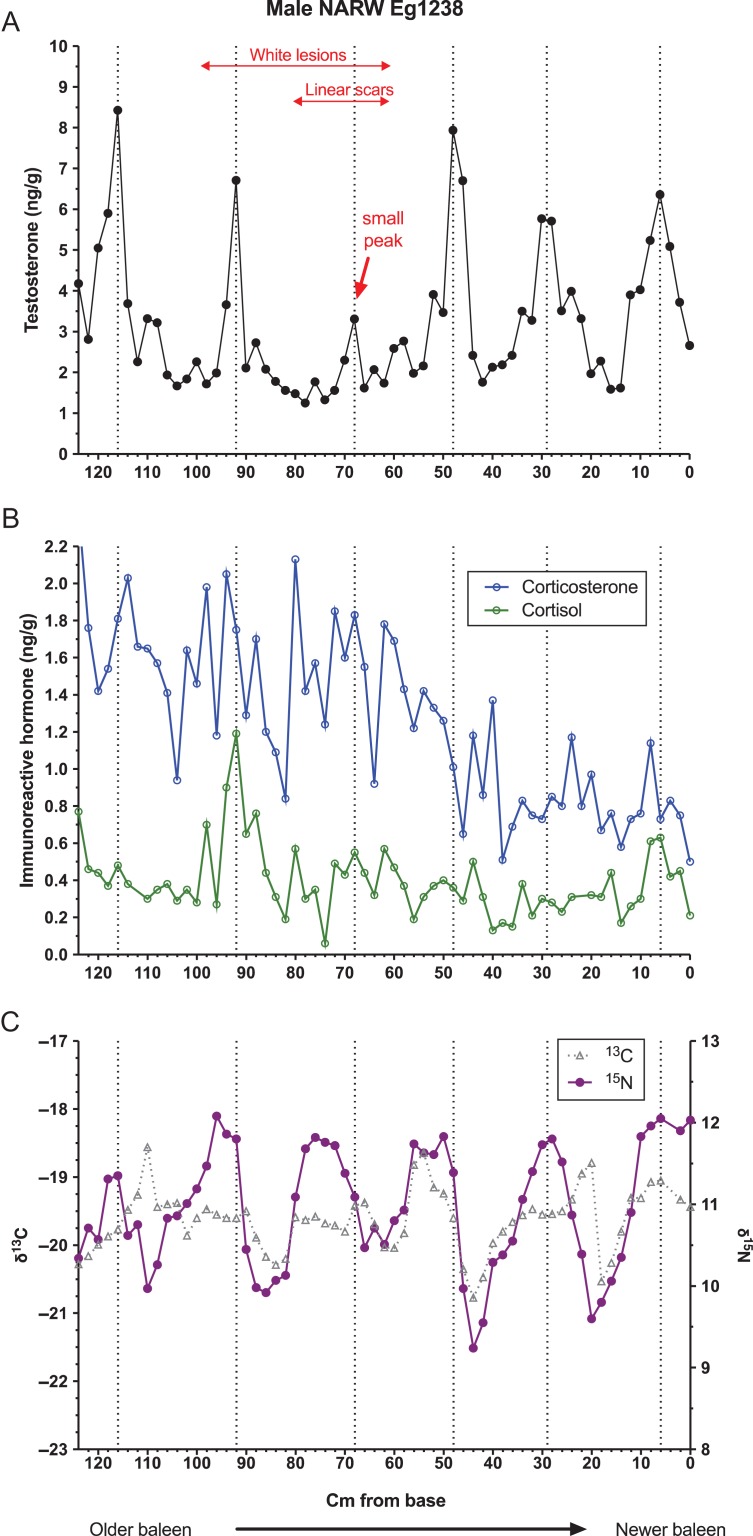
Patterns in concentrations of immunoreactive testosterone (top, A), glucocorticoids (middle, B), and ratios of stable isotopes of nitrogen and carbon (bottom, C) along the full length of a baleen plate from a single adult male North Atlantic right whale, individual Eg 1238. Dotted lines indicate locations of local testosterone maxima and minima. Arrow indicates an unusually low amplitude testosterone peak corresponding to 1998. This whale acquired linear scars indicative of entanglement in fishing gear at some point in 1998, and also acquired white lesions indicative of suspected disease during 1997–1998; estimated location along the baleen plate corresponding to these events is indicated at top.

**Figure 3:**
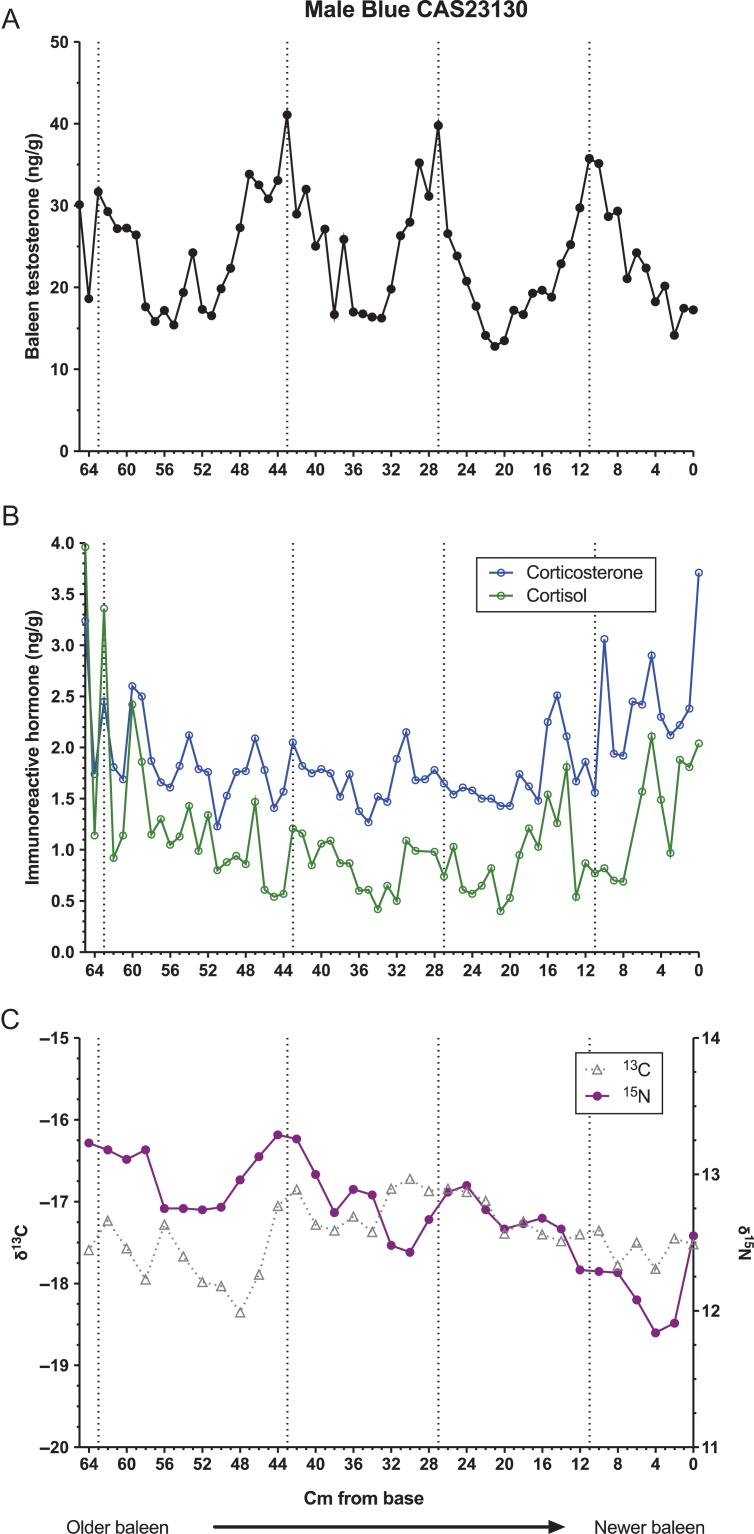
Patterns in concentrations of immunoreactive testosterone (top, A), glucocorticoids (middle, B), and ratios of stable isotopes of nitrogen and carbon (bottom, C) along the full length of a baleen plate from a single adult male blue whale, individual CAS MAM #23 130. Dotted lines indicate locations of local testosterone maxima and minima.

Baleen of all three whales contained regularly spaced areas of high testosterone content, termed ‘testosterone peaks’ (Figs 1–3, top panels). Autocorrelation analysis indicated periodicity of testosterone data in all three whales, with the period length (i.e. average cm between peaks) estimated as 17.2 cm for the bowhead, 21.0 cm for the NARW and 16.5 cm for the blue whale. Testosterone was significantly correlated with δ^15^N in the bowhead and the NARW, but not in the blue whale (Table 2).

**Table 2:** Results of statistical correlations of testosterone, cortisol, corticosterone and δ^15^N in baleen powder, measured every 2 cm (bowhead, right) or 1 cm (blue) along the full length of a baleen plate from three adult male baleen whales. Sample sizes deviate slightly from total number of samples obtained from each plate due to insufficient extract of certain samples to assay for one or both glucocorticoids, and sample spacing for stable-isotope analyses. Hormone–hormone correlations use raw data; hormone–N correlations use detrended data. Bold indicates statistical significance *P* < 0.05. NARW = North Atlantic right whale, T = testosterone, F = cortisol, B = corticosterone, N = δ^15^N

Species (individual)	T vs. F	T vs. B	F vs. B	T vs. N	F vs. N	B vs. N
Bowhead (BH3)	*r* = 0.218	*r* = 0.609	*r* = 0.106	*r* = 0.316	*r* = 0.006	*r* = 0.394
	***P*****= 0.032**	***P*****< 0.0001**	*P* = 0.303	***P*****= 0.001**	*P* = 0.953	***P*****< 0.0001**
	*n* = 97	*n* = 97	*n* = 97	*n* = 103	*n* = 93	*n* = 97
NARW (Eg1238)	*r* = 0.304	*r* = 0.003	*r* = 0.513	*r* = 0.410	*r* = 0.270	*r* = 0.240
	***P*****= 0.015**	*P* = 0.980	***P*****< 0.0001**	***P*****= 0.0009**	***P*****= 0.030**	*P* = 0.054
	*n* = 63	*n* = 63	*n* = 63	*n* = 63	*n* = 63	*n* = 63
Blue (CAS MAM 23 130)	*r* = 0.066	*r* = 0.294	*r* = 0.681	*r* = 0.270	*r* = −0.183	*r* = −0.269
	*P* = 0.603	***P*****= 0.017**	***P*****< 0.0001**	*P* = 0.156	*P* = 0.341	*P* = 0.158
	*n* = 65	*n* = 66	*n* = 65	*n* = 29	*n* = 29	*n* = 29

For the bowhead, local testosterone maxima (i.e. average peak height) averaged 8.10 ± 1.59 ng/g (mean ± standard deviation), and local minima averaged 1.36 ± 0.51 ng/g, a 6.0-fold difference. For the NARW, local testosterone maxima averaged 6.42 ± 1.82 ng/g and minima 1.58 ± 0.20 ng/g, a 4.1-fold difference. The blue whale baleen had generally higher testosterone content than baleen of the other two whales, with mean testosterone maxima of 35.22 ± 7.66 ng/g, mean minima of 14.83 ± 1.80 ng/g and a 2.4-fold difference between minima and maxima. Successive intervals between testosterone peaks decreased slightly over time in the bowhead whale, from a mean of 16.7 cm in the older half of the plate to 16.3 cm in the more recent half of the plate. In the NARW, testosterone peak spacing also declined slightly over time, from a mean of 22.7 cm in the older half of the plate to 21.0 cm in the more recent half of the plate (this calculation includes the ‘small’ testosterone peak discussed below). Similar half-plate statistical analyses could not be performed for the blue whale, which had only four testosterone peaks. However, visual inspection of the blue whale T profile indicates a potential shortening of T peak spacing over time (Fig. 3).

One of the testosterone ‘peaks’ in NARW Eg 1238’s baleen was unusually low in amplitude (Fig. 2, top panel; see arrow) and arguably this whale did not have a physiologically relevant testosterone peak in that year. This ‘small’ or ‘skipped’ testosterone cycle is estimated to have occurred in late 1998 (based on cm from base, period of 21.0 cm/year and known date of death), which is the year when Eg 1238 is thought to have experienced the minor entanglement in fishing gear and shortly after he may have experienced a disease event (i.e. white lesions that appeared during 1997–98—estimated, based on species-specific BGR and date of death, to be represented by approximately the 100–60 cm region of his baleen plate; Fig. 2).

### Glucocorticoids

All three whales had pronounced variation in baleen cortisol and baleen corticosterone (Figs 1–3, middle panels), with multiple apparent ‘peaks,’ some of which coincided with testosterone peaks and some of which occurred between testosterone peaks. The two GCs did not usually parallel each other, though occasionally both GCs were elevated coincident with a testosterone peak (e.g. first testosterone peak of the blue whale, Fig. 3), and occasionally both GCs showed prolonged or frequent elevations during the same 1–2 years (e.g. the 100–60 cm region of the NARW’s plate, estimated to have grown during approximately 1997–98; and the 12–0 cm region of the blue whale’s plate, grown during approximately 1989, within one year of death).

In pairwise comparisons of hormone data, each of the three whales exhibited a unique pattern of correlations (Table 2). In the bowhead and NARW, testosterone was positively correlated with cortisol; in the bowhead and blue whales, testosterone was positively correlated with corticosterone (Table 2). The two glucocorticoids were correlated with each other in the NARW and blue whales, but not in the bowhead (Table 2).

Similarly, autocorrelation analysis revealed differing patterns of periodicity in the glucocorticoids. The bowhead had significant periodicity for corticosterone (period = 16.5 cm, quite similar to its testosterone period of 17.2 and to average annual BGR in bowheads generally) but no detectable periodicity for cortisol. The right whale had significant periodicity for cortisol with a period length of 21 cm, identical to its testosterone period of 21 cm and again agreeing well with average annual BGR in this species. However, the right whale also had periodicity for corticosterone with a period of 31.5 cm, out of phase with the other two hormones and far longer than average BGR for this age class of NARW. The blue whale had no detectable periodicity over time for either glucocorticoid.

### Stable isotopes

The bowhead and NARW both demonstrated significant periodicity in δ^15^N and δ^13^C. For the bowhead, period lengths were 17.2 cm for both δ^15^N and δ^13^C, exactly matching this whale’s testosterone period of 17.2 cm (Fig. 1), and the two SI ratios were significantly correlated with each other (*r* = 0.302, *P* = 0.0019). The NARW had period lengths of 21.3 cm for both δ^15^N and δ^13^C, a close match to this whale’s testosterone period of 21.0 cm (Fig. 2) and again the two SI ratios were significantly correlated with each other (*r* = 0.5049, *P* < 0.0001). The blue whale did not exhibit periodicity in either δ^15^N and δ^13^C, and the two SI ratios were not correlated with each other (Fig. 3, lower panel; *r* = 0.0424, *P* = 0.8148).

## Discussion

### Do testosterone peaks in baleen represent annual cycles?

The recurrent testosterone peaks in the baleen of each male matched estimated annual BGR for each species (Lubetkin *et al.*, 2008; Lysiak, 2008; Busquets-Vass *et al.*, 2017). Further, in the bowhead and NARW, the timing of testosterone peaks matched timing of their cycles in SI ratios, and additionally testosterone was significantly correlated with δ^15^N in these two species. Cyclic SI ratios seen in baleen of bowheads and NARW are well-documented, and sightings records, hunting dates, and prey analyses indicate that these SI cycles correspond with their annual migrations between isotopically distinct summer and winter foraging areas (Best and Schell, 1996; Lee *et al.*, 2005; Lysiak, 2008; Matthews and Ferguson, 2015; Lysiak *et al.*, 2018). That is, the SI cycles in these two species are annual cycles, and therefore the matching cycles in baleen testosterone are very likely annual cycles as well. The blue whale, in contrast, did have regular testosterone peaks but did not have cyclicity in SI ratios. Recent studies by Busquets-Vass *et al.* (2017) indicate that lack of annual SI cycles in baleen may be characteristic for male blue whales in the North Pacific, likely reflecting a shortened migration route and a corresponding lack of prey-switching. Thus, we cannot confirm from SI data that the blue whale’s four testosterone peaks definitely represent annual cycles, though the good match of T peak spacing to reported BGR (Busquets-Vass *et al.*, 2017) suggests annual cyclicity. Finally, the bowhead and NARW, and possibly the blue whale, showed a slight shortening of the spacing between testosterone peaks across the length of the plate, as would be expected given the known slowing of BGR with whale age (Lubetkin *et al.*, 2008).

Though our data must be regarded as preliminary given that only three individuals were studied, the consistent patterns seen across all three species, and the excellent match to SI data in the two species known to have annual SI cycles, lead us to conclude that male baleen whales have regular annual testosterone cycles. This has long been hypothesized for these seasonally reproducing species, and also agrees well with previous reports of seasonal changes in blubber testosterone content in humpback whales sampled over one year, and hints of testosterone changes in fin, sei and minke whales sampled during summer (Kjeld, 2001; 2003; Kjeld *et al.*, 2004; Vu *et al.*, 2015). Our baleen hormone data indicates that these cycles occur repeatedly in individual males for many years in succession.

A possible alternative explanation for the regularly occurring testosterone peaks in baleen might be seasonal changes in BGR. If BGR itself changes, circulating hormones could be ‘concentrated’ into a smaller area of baleen when BGR is slow and ‘spread out’ when BGR is high, conceivably producing annual patterns of baleen hormone content that would not necessarily reflect circulating hormone concentrations. Stable-isotope studies, though, combined with matched sightings records of whale location, do not support an interpretation of large seasonal variation in BGR (see discussion in Hunt *et al.*, 2016). Minor variations in BGR are possible (Hunt *et al.*, 2016), but, given that SI data indicate regularly spaced δ^13^C and δ^15^N cycles in these and other individuals (Lubetkin *et al.*, 2008; Lysiak, 2008; Busquets-Vass *et al.*, 2017), BGR is unlikely to vary to a degree that would cause the two- to six-fold elevations in baleen testosterone content documented here. Additionally, if seasonal BGR variation were responsible for the apparent cycles of testosterone, it would presumably also cause matching periodicity in both of the GCs, which is not supported by the different patterns of one or the other GC relative to testosterone in each of the whales. Nonetheless, we recommend study of additional hormones in baleen for further investigation of this possibility, ideally including hormones that are less influenced by reproductive state (e.g. aldosterone; Burgess *et al.*, 2017) or that tend to exhibit only mild variation across the year in cetaceans (e.g. thyroid hormones; Ayres *et al.*, 2012; Rosa *et al.*, 2007). Aldosterone as well as the thyroid hormones thyroxine and tri-iodothyronine are all detectable in baleen (Hunt *et al.*, 2017b; Lysiak *et al.*, 2018). Longitudinal quantification of reproductive hormones in juvenile whales may also help clarify this question, since juveniles presumably would not have the pronounced annual androgen cycles seen here in adult males. This latter approach is complicated, however, by the fact that age of male sexual maturity is not definitively known for many mysticete whales. For example, male NARW are not known to father calves before age 15, perhaps due to inability to compete with older males, but are suspected to reach sexual maturity at ages 9–10 (Frasier *et al.*, 2007).

The dramatic oscillations in testosterone documented here suggest that future studies of cetacean endocrinology may need to incorporate the possibility that adult males may have quite low testosterone outside of the putative breeding season. For example, efforts to determine the sex of unknown-sex individuals via androgen:estrogen ratios, and/or to discriminate age class (juvenile vs. adult) using absolute values of androgens, may only be advisable in those months of the year when adult males actually do have elevated testosterone. Indeed such efforts have produced varying results, with gonadal steroids correctly identifying sex and male age class for some species in some seasons (e.g. NARW in August and September, fecal samples, Rolland *et al.*, 2005) but not in other species or in other seasons (e.g. common minke whale in May–September, plasma samples, Kjeld *et al.*, 2004). Similarly, efforts to validate novel sample types via predictions of expected sex or age-class differences in hormones should be employed with caution. A novel sample type collected in the non-breeding season may not necessarily show expected endocrine differences between the sexes or between maturity classes (e.g. male and female NARW with similar respiratory vapor androgen content in spring, Burgess *et al.*, 2018). We recommend further studies on seasonal patterns of all the gonadal steroids, in both sexes and all age classes, to determine whether ratios and absolute values of gonadal steroids can be used to identify age class and sex in large whales in all months of the year or only in certain seasons.

### Which glucocorticoid is most informative for stress physiology?

It has long been assumed that cortisol is the dominant circulating hormone in mysticete whales and that corticosterone, if it is detectable at all, would only parallel cortisol patterns (see discussion in Hunt *et al.*, 2017a). In this study, as in other recent baleen hormone studies (Hunt *et al.*, 2017a,b; Lysiak *et al.*, 2018), both these assumptions have faltered. All three whales generally had more immunoreactive corticosterone than cortisol in baleen, and the two hormones did not always parallel one another. The bowhead showed no significant correlation of cortisol to corticosterone, while the NARW and blue did have significant correlations of the two GCs over time. Recent data from terrestrial mammals indicates that the two GCs may respond quite differently to stressors and that they may not always correlate within or among individuals (Koren *et al.*, 2012). Yet, there are indications that the two GCs occasionally synchronize, especially during times of more severe stress (i.e. the ‘emergency life history state’; Romero and Wingfield, 2015). In prior studies of baleen hormones in females, the two GCs show different patterns during ‘baseline’ or non-stressful periods (e.g. intercalving intervals of female NARW; Hunt *et al.*, 2017a), but can show simultaneous elevations during those reproductive states known to entail high energetic burden, i.e. documented pregnancies (NARW, baleen; Hunt *et al.*, 2017a). Similarly, both GCs show elevations during confirmed severe stressors such as chronic entanglement in fishing gear (NARW, Lysiak *et al.*, 2018), repeated wounding (southern right whale, *Eubalaena australis*; Fernández Ajó *et al.*, 2018), and prolonged chronic illness (humpback whale; Hunt, unpublished data). In this study, there was only one known case of prolonged chronic stress independently confirmed through sightings data, the NARW that experienced possible disease and a definite entanglement in 1997–98. In this individual, both GCs were indeed elevated above presumed baseline in baleen estimated to have been grown during 1997–1998, and the two GCs were significantly and positively correlated. Though these patterns are preliminary, our results, combined with the aforementioned studies, suggest that simultaneous elevations in both GCs in baleen (as opposed to just one or the other of the GCs) may be a potential marker of prolonged or severe stress.

### Relationship of testosterone to glucocorticoids

Interrelationships of testosterone to the glucocorticoids are of particular interest from a conservation and management perspective, for two reasons: periods of high testosterone can increase stress (i.e. mating-related stressors can affect efforts to quantify cumulative stress), and conversely, periods of chronic stress can interfere with reproduction (reviewed in Romero and Wingfield, 2015). In this study, correlations between testosterone and the glucocorticoids were highly variable across the three individuals. Testosterone was significantly correlated with both glucocorticoids in the bowhead, with cortisol only in the NARW, and with corticosterone only in the blue whale. Such variation would be expected if the different species, or different individual whales, experience different patterns of breeding-related stressors *versus* non-breeding-related stressors. Notably, all three whales had at least one testosterone peak that exactly coincided with an elevation in both glucocorticoids, for example, the seventh testosterone peak in the bowhead whale, the second testosterone peak in the NARW, and the first testosterone peak in the blue whale. This suggests that breeding-season-related stress did sometimes affect all three individuals and species, yet all three whales also had other testosterone peaks that had low or no associated glucocorticoid peaks. Our interpretation is that these variable patterns may indicate that ‘some breeding seasons are more stressful than others,’ i.e. there may be random annual variation in breeding success and in inter-male competition.

The reduced testosterone peak seen in Eg 1238 in late 1998 may indicate potential influences of stress on reproduction. Of all the three whales, this was the only case of a ‘missing’ or ‘short’ (low amplitude) testosterone peak manifest as a gap in the otherwise regular spacing of high amplitude testosterone peaks. Interestingly, this gap in high amplitude peaks occurred near the end of, or immediately after, the only confirmed episode of stress (i.e. Eg 1238’s entanglement and potential disease event). In other mammals, prolonged stress is well-documented to inhibit reproduction, and not infrequently has continued negative impacts on reproduction for months or years after the stressor (e.g. Buck *et al.*, 2007; reviewed in Romero and Wingfield, 2015). In female whales, entanglement is suspected to cause long-term impacts on reproduction; for example, entangled female NARW, even if they shed the entanglement, tend to delay the next calving and in some cases are not observed to calve again even after years (Knowlton and Kraus, 2001; Van der Hoop *et al.*, 2017). It has been unclear whether stress might similarly affect reproduction in male NARW. Male NARW do not have to bear the costs of pregnancy or lactation, yet breeding may be costly for them nonetheless; NARW use a scramble-competition mating system that involves intense and prolonged interactions between dozens of males that compete for positioning next to a single central female (Ginsberg and Huck, 1989; Mate *et al.*, 2005). In fact, NARW testes are the largest ever recorded for any animal species (Brownell and Ralls, 1986) indicating intense sperm competition among males (Ginsberg and Huck, 1989; Mate *et al.*, 2005). These traits suggest an energetic cost for reproduction in male NARW, such that it may be adaptive to reduce or ‘skip’ investment in reproduction in a given year if body condition is poor, but there has not before been a method for assessment of potential impacts of stress on male reproduction. Though Eg 1238 represents only a single case study, his testosterone profile suggests that stressful events could indeed inhibit reproductive performance in male right whales. Given recent high rates of entanglement and mortality in NARW, combined with record-low calving numbers (Pettis and Hamilton, 2016, 2017; NOAA, 2018; Meyer-Grutbrod and Greene, 2018), the potential long-term costs of stress on reproduction in both sexes in this species must be regarded as a priority for future research. Further study of baleen from cases of known cause of death of breeding-age adults, especially comparison of acute trauma cases (i.e. shipstrike) to chronic-stress cases (i.e. entanglement, prolonged disease, etc.) would be especially fruitful.

We caution that the exact chemical identities of the immunoreactive compounds detected by the testosterone, cortisol and corticosterone assays have not been definitively determined. The data presented here may conceivably represent not pure parent hormones but rather some mix of metabolites—some of which may even be derived from non-target hormones, as can occur with fecal hormone analyses (see discussion in Hunt *et al.*, 2006). Further studies employing high-performance liquid chromatography (HPLC) or mass spectrometry will be necessary to identify the exact chemical identity of putative hormones in baleen. Even so, it still will not be clear what parent compound such metabolites derive from, since classic pituitary-hormone challenge studies (ACTH challenges, etc.) and radiolabel infusions are not possible in mysticete whales. However, the studies discussed above indicate that baleen progesterone does reflect reproductive state in females; that baleen GCs do correlate with known, identifiable chronic stress and reproductive states of high energetic burden; and in this study, that baleen testosterone exhibits the annual elevations that are predicted for adult males of seasonally breeding species. We encourage investigation of the chemical identity of immunoreactive baleen steroids, but our present data suggest that baleen testosterone and baleen GCs likely do represent a retrospective record of gonadal and adrenal activity of individual males over years.

## Conclusions

Our data, though limited to three individuals, indicate that adult male mysticete whales likely experience regular, repeated annual elevations in testosterone, and furthermore that baleen glucocorticoids may be useful for identification of episodes of stress. Further study of testosterone patterns in baleen of males, and interrelationships with the adrenal glucocorticoids, could prove helpful for clarifying age of sexual maturity in males (as opposed to age of documented paternity, which likely lags behind age of sexual maturity by several years); identifying season and potentially location of breeding; potential occurrence of reproductive senescence; and delineating the impacts of natural and anthropogenic stressors on male reproduction. Combined with recent reports on patterns of baleen progesterone in females, and with a growing body of evidence that both baleen glucocorticoids elevate during a variety of types of chronic stress, the technique of baleen hormone analysis appears to have widespread applicability to studies of reproduction and stress physiology in all age classes and both sexes of mysticete whales.
